# Serological Immunity to Smallpox in New South Wales, Australia

**DOI:** 10.3390/v12050554

**Published:** 2020-05-18

**Authors:** Valentina Costantino, Mallory J. Trent, John S. Sullivan, Mohana P. Kunasekaran, Richard Gray, Raina MacIntyre

**Affiliations:** 1 Biosecurity Program, Kirby Institute, Faculty of Medicine, University of New South Wales, Sydney, NSW 2052, Australia; mjtrent@protonmail.com (M.J.T.); mohanapk@protonmail.com (M.P.K.); rainam@protonmail.com (R.M.); 2 Central Clinical School, University of Sydney, Sydney, NSW 2052, Australia; john.sullivan@unsw.edu.au; 3 School of Medical Sciences, University of New South Wales, Sydney, NSW 2052, Australia; 4 Surveillance Evaluation and Research Program, Kirby Institute, Faculty of Medicine, University of New South Wales, Sydney, NSW 2052, Australia; rgray@kirby.unsw.edu.au

**Keywords:** smallpox, vaccine immunity, response planning, population immunity

## Abstract

The re-emergence of smallpox is an increasing and legitimate concern due to advances in synthetic biology. Vaccination programs against smallpox using the vaccinia virus vaccine ceased with the eradication of smallpox and, unlike many other countries, Australia did not use mass vaccinations. However, vaccinated migrants contribute to population immunity. Testing for vaccinia antibodies is not routinely performed in Australia, and few opportunities exist to estimate the level of residual population immunity against smallpox. Serological data on population immunity in Australia could inform management plans against a smallpox outbreak. Vaccinia antibodies were measured in 2003 in regular plasmapheresis donors at the Australian Red Cross Blood Service from New South Wales (NSW). The data were analysed to estimate the proportion of Australians in NSW with detectable serological immunity to vaccinia. The primary object of this study was to measure neutralising antibody titres against vaccinia virus. Titre levels in donor samples were determined by plaque reduction assay. To estimate current levels of immunity to smallpox infection, the decline in geometric mean titres (GMT) over time was projected using two values for the antibody levels estimated on the basis of different times since vaccination. The results of this study suggest that there is minimal residual immunity to the vaccinia virus in the Australian population. Although humoral immunity is protective against orthopoxvirus infections, cell-mediated immunity and immunological memory likely also play roles, which are not quantified by antibody levels. These data provide an immunological snapshot of the NSW population, which could inform emergency preparedness planning and outbreak control, especially concerning the stockpiling of vaccinia vaccine.

## 1. Introduction

Smallpox was introduced into Australia with the arrival of the First Fleet in 1787, resulting in severe outbreaks among the indigenous population [1,2]. Although smallpox was endemic in most countries by 1920, it never became an established endemic disease in Australia. Australia’s geographical remoteness protected it from all but a few ongoing importations of smallpox, which were effectively controlled by quarantine measures at seaports. Most settlers in Australia likely had immunity to smallpox through natural infection or vaccination in Europe or elsewhere [3]. The last major outbreak of smallpox in Australia was in May 1913, when variola minor, a milder form of the disease, was imported from Canada, resulting in 2398 cases and four deaths in New South Wales [4]. The last documented case of smallpox in Australia occurred in 1938 [5].

The re-emergence of smallpox is now an increasing and legitimate concern [6]. Advances in synthetic biology have enabled de novo virus synthesis [7]. Canadian researchers synthesised a closely-related orthopoxvirus and published their methods in 2018 [8]. We previously showed that smallpox reintroduction into Australia, where an estimated 17% of people live with moderate to severe immunosuppression, could result in high transmission and a 45% case fatality rate [9].

Although Australia never had a universal smallpox vaccination program, several states implemented compulsory vaccination programs in the mid-19th century, including South Australia, Western Australia, Victoria, and Tasmania [4]. About 30% of children born in Australia between 1860 and 1910 were vaccinated against smallpox, but by 1923, the proportion of infants vaccinated against smallpox decreased to less than 10% [3]. From 1960 to 1976, with the World Health Organization’s (WHO) push for global smallpox eradication, an estimated 5 million smallpox vaccination doses were administered in Australia through supplementary immunisation activities [10]. Vaccination coverage in Australia was relatively low compared to countries such as the U.S., where more than 90% of Americans born before 1971 were vaccinated against smallpox [11]. In the absence of a past universal vaccination program and combined with a low endemicity for smallpox, we previously estimated that among the current population in Sydney, Australia, only 30% of people born prior to 1980 (mostly immigrants who were vaccinated in their country of origin) have been vaccinated [9].

Studies of the duration of immunity after smallpox vaccination have yielded mixed results. The U.S. Center for Disease Control (CDC) suggests immunity wanes to almost zero 5–10 years post-vaccination [9,12]. However, a duration of protection of >20 years was consistently seen among 16 retrospective cross-sectional studies [13]. Even if serological immunity wanes, past vaccination is thought to protect against fatal infection, following findings that human memory B and T cells can be maintained for life in the absence of antigenic re-exposure [14]. Several factors were shown to affect residual immunity to smallpox, including sex, age at time of vaccination, ethnicity, gene polymorphisms, and type of smallpox vaccine received [15,16,17,18,19,20]. There is little contemporary serological data on population immunity in Australia, but any data could inform management plans against a smallpox outbreak [21].

This study had two aims: (1) to estimate the proportion of Australians who have detectable neutralising antibodies against vaccinia virus based on serological data from 2003 and (2) to model the waning of immunity over time to project current levels of immunity.

## 2. Methods

### 2.1. Aim 1

#### 2.1.1. Study Population and Recruitment

A study was conducted in 2003 to estimate the proportion of Australians in New South Wales (NSW) with detectable serological immunity to smallpox. We recruited subjects from NSW who were regular plasmapheresis donors at the Australian Red Cross Blood Service (ARCBS) in the year 2003. The study participants ranged from age 16 to 76 years at the time of donation and their smallpox vaccination and infection history was unknown. The data were not analyzed at the time, but in 2019 author J.S., who was at the Australian Red Cross Blood Service (ARCBS) in 2003, made the data available for analysis with approval from ARCBS.

To recruit participants, the Apheresis Medical Officer approached donors and informed them of the study, and donors had the option to accept or decline to participate. Participating donors provided written consent prior to data collection. A 20–30 mL blood sample was collected from participants at the time of donation, along with data on donor date of birth and sex. The study protocol and consent documents were approved by the Human Research Ethics Committee at the Australian Red Cross Blood Service.

#### 2.1.2. Estimation of Neutralising Antibody Titre against Vaccinia Virus

The primary object of this study was to measure neutralising antibody titre against vaccinia virus. Titre levels in donor samples were determined by the plaque reduction assay. Serum samples were heat-inactivated for 30 min at 56 °C. Initial 1:5 dilution of serum was performed in RPMI + 0.2% BSA (firstly developed at the Roswell Park Memorial Institute, hence the name), followed by five to eight further 2-fold serial dilutions. To 600 µL of serum dilution (or medium alone for control), 600 µL of vaccinia virus (NYCBH strain) was added, at 800 PFU/mL, in RPMI + 0.2% BSA. Serum and virus mixtures were incubated at 37 °C for 60 min. Then 500 µL was added to duplicate wells (6-well plates) of serum-free RPMI-washed BSC-1 cell monolayers. Monolayers were incubated for 60 min at 37 °C, with regular rocking of the plates to allow even distribution of the virus solutions and to prevent dry patches forming. Subsequently, 3 mL/well of RPMI + 2% FCS (Fetal calf serum) was added, and monolayers were incubated for 36–48 h. Then the medium was removed and stained with 1 mL/well of crystal violet stain (0.5% in methanol) for 10 min, before washing with water. Virus plaques were counted by visual inspection, and residual virus not neutralised by serum was compared with total virus (serum-free incubations). Results are expressed as percent neutralisation.

#### 2.1.3. Statistical Analyses

A total of 179 blood donors were recruited into the study and provided a serum sample. No information on previous vaccination history, refusal rates, or sample size calculation was available. All data were cleaned and deidentified prior to analysis. Statistical analysis and figure preparation were performed using Stata version 14 [22]. Geometric mean titres (GMT) were computed for each age group included in the study. Student’s *t*-test was used to compare geometric mean titres between participants that were less than 40 years of age and participants that were 40 years or older. *p* ≤ 0.05 was considered statistically significant. Participants with an antibody titre of 1:32 or higher were considered seropositive based on the results from Mack et al. [23], which showed that smallpox patients’ contacts who had neutralising titres <1:32 against vaccinia virus were more susceptible to smallpox infection (20% of contacts infected) than contacts with pre-existing antibody titres ≥1:32 (zero contacts infected). No contacts that had a titre of 1:32 or higher developed smallpox.

Based on these older data from the prospective study conducted during the endemic period of smallpox, the 1:32 cut-off titre has been widely accepted as a reasonable biomarker of protective immunity and was also used in other clinical studies [24]. Based on those results, we considered people who had neutralising titres >1:32 seropositive, i.e., previously vaccinated.

### 2.2. Aim 2

To estimate current levels of immunity to smallpox infection, we modelled the decline in GMT over time since vaccination. We first searched for published literature reporting the GMT level just after vaccination. We then used the 2003 serological results of antibody titre levels found in NSW and estimated the possible time since vaccination to calculate the rate of decline in GMT, assuming an exponential model.

#### Data/Estimates for Projection of Waning Neutralising Antibody Titre

We found four different studies using the vaccinia-specific plaque reduction serum neutralisation assay to measure the level of neutralising antibody titre [25,26,27,28] pre- and post-re-vaccination for smallpox protection. However, only one of those studies used the Dryvax vaccine [28], a first generation vaccine used during the eradication period. In that study, 1124 civilians were vaccinated with Dryvax and their vaccinia-specific antibody titres were measured before vaccination and one month after vaccination. They reported results by age, number of previous smallpox vaccinations, and time since last vaccination. To project GMT level over time (years) since vaccination, we compared results from the Australian samples with the results from the previously mentioned study [28] for GMT levels following vaccination.

The time since previous vaccination in the Australian sample was estimated using estimated past vaccination history based on age at the time of testing in 2003. Smallpox vaccination ceased in 1980, 23 years before these samples were taken. Therefore, the shortest period since vaccination in this population would have been 23 years. Since the last vaccinations in Australia occurred from 1960 to 1976, the longest period of time since vaccination in this population would have been approximately 27 to 40 years [29]. However, people who migrated to Australia could have been vaccinated in their country of birth as infants. Therefore, for each age group, we assumed the possibility that everyone was vaccinated at one year old or in the previous 23–40 years. When calculating the number of years since vaccination in the case of being vaccinated at one year old, we considered the mean age for each age group. For the 30–39 years age group, the mean point is 35 years old, so being vaccinated at one year old for this age group would mean being vaccinated 34 years prior. The same was performed with the other two age groups. For the 40–49 and 50+ years age groups, vaccination at one year old meant being vaccinated 44 and 64 years prior, respectively. 

Prior studies suggested that after smallpox infection or vaccination, the magnitude of the antibody, as well as T cell responses, wane exponentially over time [11,14,30]. To obtain an exponential decay function of the GMT levels following vaccination, we first calculated the decreasing annual rates of GMT for each scenario using the two GMT values we had in the two different time points following vaccination, then we projected it in time.

## 3. Results

### 3.1. GMT Levels in the Australian Population

A total of 179 plasmapheresis donors were recruited to participate in the study. Of these, 49 (27%) were less than 30 years of age in 2003, 33 (18%) were between the ages 30 and 39, 45 (25%) were between the ages 40 and 49, and 52 (30%) were 50 years of age or older; 100 (56%) participants were male and 79 (44%) were female. The average age of donors did not vary significantly between men and women (*p* = 0.21).

**Figure 1:**
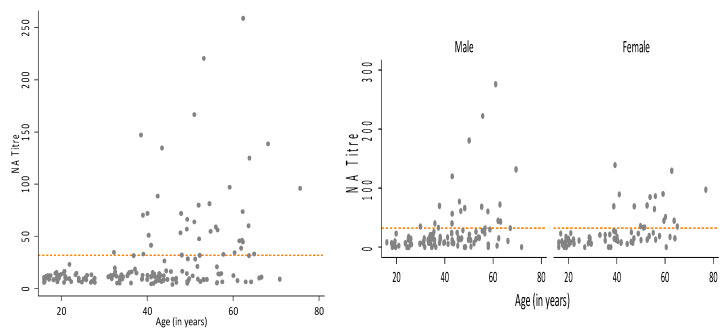
Plot of anti-vaccinia neutralising antibody titre by age, for total sample (*n* = 177) and by sex (*n*_male_ = 100, *n*_female_ = 77).

Anti-vaccinia neutralising antibody titres were available for a total of 177 out of 179 participants. Figure 1 shows neutralising antibody titres plotted against age, overall, and by sex. GMT was significantly higher in donors 40 years and older compared to donors less than 40 years of age in 2003 (*p* < 0.001). GMT did not vary significantly by sex (*p* = 0.62).

**Figure 2:**
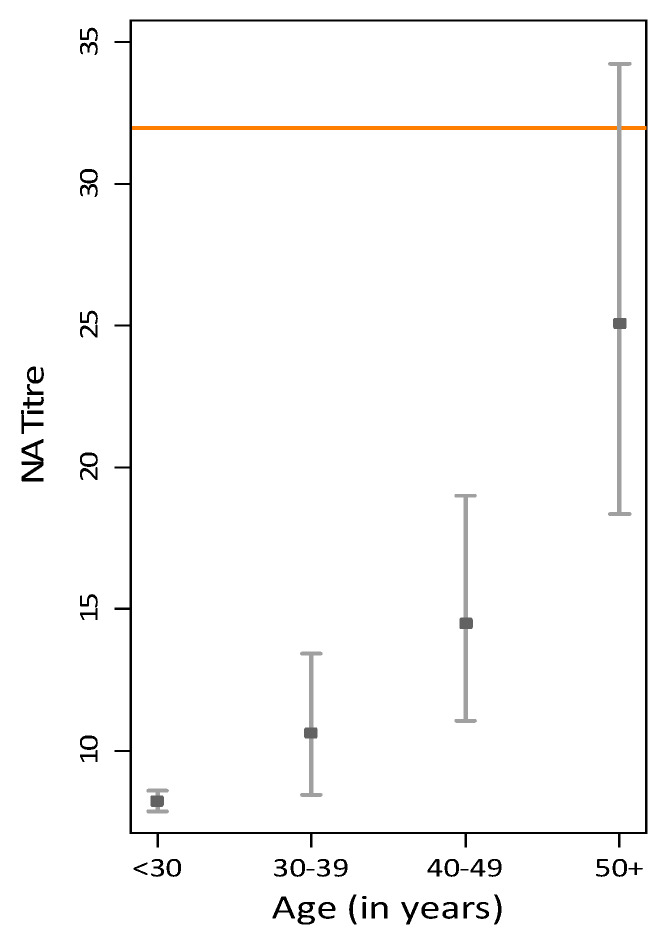
Geometric mean titres with 95% confidence interval (CI) for anti-vaccinia neutralising antibody by age group, including seronegative (horizontal line is the level considered seropositive, >32).

Geometric mean titres (GMT) and 95% confidence intervals for anti-vaccinia neutralising antibodies in each age group are shown in Figure 2. For donors less than 30 years old, the GMT was 8.21 (95% confidence interval (CI), 7.86–8.58). Donors between the ages 30 and 39 years had a GMT of 10.63 (95% CI, 8.43–13.42). For donors aged 40–49 years, the GMT was 14.49 (95% CI, 11.05–18.99). Donors aged 50 years and older had a GMT of 25.07 (95% CI, 18.35–34.25). When applying the seroconversion cut-off at >32, of donors less than 30 years old, 0% were seropositive, whereas 9%, 24.5% and 48% were seropositive in the age groups 30–39, 40–49, and 50+ years, respectively. The results are shown in Table 1. When restricting the analysis to only seropositive donors (21.5% of the total sample), we found that the GMT by age group is 56 for those aged 30–39 years, 62 for donors aged 40–49 years, and 68 for donors aged 50 years or older.

### 3.2. Projection of Waning Neutralising Antibody Titre over Time Since Vaccination

Assumptions regarding time since vaccination for the Australian sample analysed are shown in Table 2. In the last two columns, we show results for two different scenarios by lower and upper limits of the possible time since vaccination for each age group. The GMT level following vaccination is shown in the second column of Table 2.

**Figure 3:**
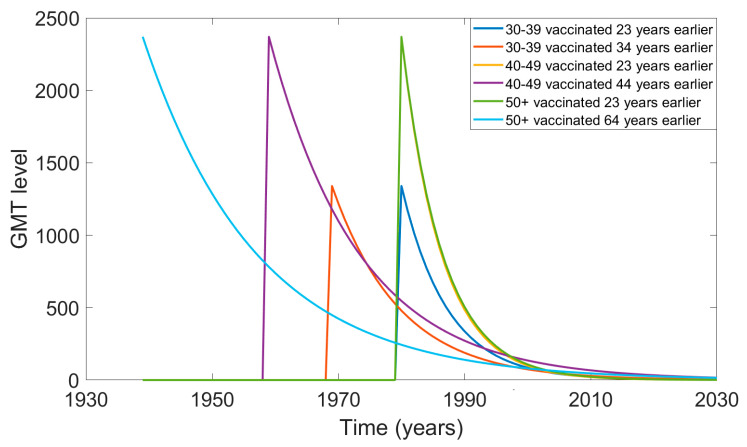
Projected decline in GMT level over time for the two scenarios for time since last vaccination in each age group.

The GMT values over time since vaccination are shown in Figure 3. For the first scenario (column 3, Table 2), we assumed each age group was vaccinated 23 years before 2003 (in 1980). For the second scenario (last column, Table 2), in which we assumed people were vaccinated as infants, the graph shows vaccine uptake in 1969, 1959, and 1939 for the 30–39, 40–49, and 50+ years age groups, respectively.

For the 30–39 years age group that was found with some immunity in 2003, their level of GMT was below the threshold of 32 by 2010. For the 50+ years age group, in the scenario of being vaccinated in 1980, their GMT level would have fallen below 32 by 2008, whereas in the scenario of being vaccinated 64 years before 2003, their GMT level would have been protective until 2017. In the scenario of being vaccinated 23 years earlier, the 40–49 years age group showed no protective immunity after 2008. In the scenario of being vaccinated 44 years earlier (in 1959), they would have protective immunity until 2021.

## 4. Discussion

Ascertaining seroprevalence to vaccinia in Australia is useful given the threat and potential impact of the re-emergence of smallpox and the dearth of serological data [31]. The results of this study suggest that residual immunity to vaccinia virus is minimal in the Australian population. This has important implications for emergency preparedness planning and outbreak control, especially concerning the stockpiling of vaccinia vaccine [31,32]. Generally, immunity wanes with time since vaccination [33,34], and smallpox is no exception [35]. However, there is no consensus on the exact duration of protection against smallpox from vaccination or from natural infection [13], and past vaccination is likely to protect against fatal infection [14].

In countries where universal smallpox vaccination was practiced, over 20% of the population may have residual vaccine-induced immunity, but its degree and duration are uncertain [9]. There is a wide range of estimates for the duration of protection from smallpox infection or vaccination. Estimates were mostly obtained from vaccine trials and observational studies of previous outbreaks, none of which were primarily designed to study the duration of immunity [11,13]. In studies that used Dryvax, a significantly lower plaque reduction neutralisation titre was observed compared with using variola as the antigen [36]. Understanding the antigenic differences between virus and vaccine neutralisation titres could result in improved estimates of the duration of immunity as a measure of protection.

Evidence exists of long-lasting immunity given the persistence of neutralising antibodies (Nabs) after smallpox vaccination for at least 20 years [11,37,38,39]. Of the total antibodies produced in response to smallpox vaccination, Nabs significantly contribute to immunological memory [40]. In a longitudinal study in the U.S., antibody titres persisted and remained relatively constant for up to 88 years after vaccination [24]. Hammarlund et al. found that anti-vaccinia antibody responses persisted up to 75 years post-vaccination [11]. Similarly, several adults in our study were still considered seropositive for vaccinia, suggesting that antibody responses can persist for decades even in the absence of natural boosting. Although the majority of adults sampled in our study did not have residual immunity to vaccinia, this can be explained by the low vaccine coverage in Australia, which never had universal vaccination [9]. The number of previous doses received also influences the GMT level [28] and this could explain why, in Australia, the GMT levels found in the 2003 sample were very low compared to the U.S. population. These studies suggest a longer duration of immunity following vaccination than the three to five years that is assumed in smallpox guidelines, and question whether boosting at regular intervals is required [13].

Smallpox vaccination is also highly protective against other orthopoxvirus infections. The number of monkeypox cases has been increasing in the last two decades, with travel-related cases occurring in the U.K. and Singapore [41]. A possible explanation for the resurgence of monkeypox could be waning immunity due to smallpox vaccine cessation, resulting in a largely susceptible population [42].

The limitations of this study include a small sample size and only a single cross-sectional snapshot of seroprotection. These were the only available data, and vaccinia serology is no longer routinely performed in laboratories, making this an important opportunity to study immunity to smallpox in Australia. In addition, with the eradication of smallpox in 1980, it may be impossible to definitively establish immunological correlates of protection against the disease in humans. The only available titre cut-off values for seroconversion were >1:20 [43] and >1:32 [23], reported during the period of endemic smallpox circulation, which may not be relevant to the contemporary population [13]. From observations in the field and in trials, those who have immune correlate levels below the cut-off threshold for Nab levels also appear to still have protection [44,45]. With more than 200 proteins, the smallpox vaccine based on vaccinia virus strains is relatively more complex compared to contemporary vaccines [46]. A wide range of potential antigenic epitopes could yield a varied and diverse immune response and could provide multiple options for long-lived immunity. Although humoral immunity is protective against orthopoxvirus infections, cell-mediated immunity and immunological memory likely also play roles, which are not quantified by antibody levels. In the era of smallpox circulation, there was also the possibility for subclinical infections to occur after vaccination, which could boost the immune response and provide an inaccurate view of the duration of protection from the vaccine [11]. Thus, our estimate of the proportion of Australians that are seropositive against vaccinia may not accurately reflect the proportion that are clinically protected against smallpox. We found that the oldest group had the highest level of neutralising antibody titre, which is likely a consequence of vaccine response and immunity level being correlated to the age of the vaccinee and the number of previous vaccinations [13]. In Australia, smallpox was never endemic, mass vaccination was never used, community vaccination was not performed after 1980, and the last case was reported in 1938 [9]. As such, the majority of the younger age groups would never have been vaccinated. We previously estimated that no one born after 1980 was vaccinated, and only 30% of the total population born before 1980 (people 35–69 years of age) had been vaccinated [9]. This explains the higher seropositivity in older age groups. We assumed an exponential decline in immunity, consistent with other modelling studies where the loss of immunity is assumed to be at a constant rate [47,48].

Due to the lack of data on vaccination status and time since vaccination of the participants, we used a number of assumptions in estimating the decreasing rate of the antibody level over time since vaccination. However, we conducted sensitivity analysis on the possible range in the interval for the time since vaccination, and all scenarios confirm the main conclusion of the work—that residual anti-smallpox immunity in the Australian population is extremely low.

A recent systematic review showed evidence of long-term protection of more than 20 years in 16 retrospective cross-sectional studies, where the lowest estimated duration of protection was 11.7 years [13]. We previously estimated that in Sydney, the impact of residual vaccine-induced immunity was virtually absent, and that population immunosuppression had correspondingly increased, leaving the population more vulnerable than ever to re-emergent smallpox [9]. This study provides confirmation through available serological data that as of 2019, almost 40 years after smallpox eradication, there is very little residual immunity in Australia.

## Figures and Tables

**Table 1:** Geometric mean titres (GMT) for anti-vaccinia neutralising antibody by age group for the total sample and restricted to the people considered seropositive (titre ≥ 32), with 95% confidence interval (CI).

Age (Years)	GMT Total Sample	n	% Seropositive	GMT for Seropositive	95% CI
<30	8.21	0	0.00%	-	-
30–39	10.63	3	9.09%	56.3	(6.84, 463.62)
40–49	14.49	11	24.44%	61.74	(48.09, 79.26)
50+	25.07	24	48.00%	68.33	(52.87, 88.31)

**Table 2:** Age-specific GMT levels at different estimated times since vaccination.

Age (Years)	GMT Following Vaccination (First Year after Vaccination) [28]	GMT at the Shortest Time Since Vaccination t = 23 Years	GMT at the Longest Time Since Vaccination (at 1 Year Old) t = 34, 44, and 64 Years Depending on the Age Group
30–39	GMT (1) = 1340	GMT (23) = 56	GMT (34) = 56
40–49	GMT (1) = 2370	GMT (23) = 62	GMT (44) = 62
50+	GMT (1) = 2370	GMT (23) = 68	GMT (64) = 68
